# Gender and Age Differences and the Trend in the Incidence and Prevalence of Dementia and Alzheimer's Disease in Taiwan: A 7-Year National Population-Based Study

**DOI:** 10.1155/2019/5378540

**Published:** 2019-11-11

**Authors:** Chih-Ching Liu, Chung-Yi Li, Yu Sun, Susan C. Hu

**Affiliations:** ^1^Department of Public Health, College of Medicine, National Cheng Kung University, No. 1, University Road, Tainan City 701, Taiwan; ^2^Department of Public Health, College of Public Health, China Medical University, Taichung 404, Taiwan; ^3^Department of Neurology, En Chu Kong Hospital, No. 399, Fuxing Road, Sanxia District, New Taipei City 23702, Taiwan

## Abstract

**Background:**

Very few nationwide studies have focused on the variations in the incidence and prevalence of dementia and Alzheimer's disease (AD) in Asian countries. This study aims to describe the gender and age differences in the incidence and prevalence of dementia and AD in Taiwan.

**Methods:**

The data on dementia and AD were acquired from the Taiwan National Health Insurance Research Database from 2004 to 2010. The sex and age-specific rates were standardized, and the differences of gender and age on dementia or AD were assessed using Poisson regression analysis.

**Results:**

Over seven years, the prevalence of dementia and AD significantly increased from 4.7 to 7.6 per hundred people (*β* = 0.0784, *p* < 0.0001) and 2.3 to 3.5 per hundred people (*β* = 0.0696, *p* < 0.0001), respectively. However, the incidence of both dementia and AD decreased but not significantly from 10.9 to 10.7 and 4.9 to 4.6 per thousand person-years, respectively. Noticeably, both incidence and prevalence increased with age and were higher in women than in men.

**Conclusions:**

The standardized incidence rates of dementia and AD are much lower than the data reported in some studies from Europe, the US, and Japan. Further studies are warranted to explore which factors are associated with the differences in the incidence of dementia and AD in Taiwan.

## 1. Introduction

Dementia has been considered as a public health priority as it commonly leads to increase in terms of disability, mortality, and cost [1, 2]. The most common type of dementia is Alzheimer's disease (AD) which accounts for nearly 50%–70% of dementia in the world [3]. More than 90% of dementia and AD onset occur in individuals aged 65 years and older [3, 4]. However, not all studies have distinguished AD from all type of dementia.

Due to the rapidly ageing population in the world, several studies have reported an increasing trend in the prevalence of dementia [5–11] and AD [7, 8, 10] over the past decades. Some studies have also reported increased trends in the incidence of dementia [8, 9, 12] or AD [8, 13, 14] over time, while other studies in high-income countries have shown no change [15–18] or even a decrease [6, 19, 20] in the past decades. These inconsistent results indicate the risk of dementia and AD is variable; however, limited nationwide studies on the secular trend of the incidence and prevalence of dementia and AD have been conducted in Asia [8–11, 14].

Previous studies revealed that the incidence and prevalence of dementia and AD vary across race/ethnic groups. For all types of dementia, recent meta-analysis found that the age-gender-standardized incidence and prevalence of dementia in Western Europe were 17.3 per 1,000 person-years and 6.8 per 10^2^ people, respectively, which were higher than the figures reported in East Asia [2]. For AD, a meta-analysis reported that the crude incidence and prevalence of AD among elderly adults in Europe were 11.1 per 1,000 person-years and 5.1 per 10^2^ people, respectively [21], which is also higher than some of East Asian countries such as China and Taiwan [10, 22]. However, most of the above Asia data were derived from regional community surveys or selected institutions [2, 10, 22], which are subject to potential nonrepresentativeness.

Taking Taiwan for example, to the best of our knowledge, only two national studies have been conducted in Taiwan to investigate the incidence of dementia [9] and AD [14]. One national study in Taiwan was medical claim-based and reported the age-adjusted incidence of dementia was 9.6 per 1,000 person-years∼11.1 per 1,000 person-years from 1997 to 2003 among people aged 65 and over. However, the secular trends in sex-stratified dementia incidence rate in the same periods were not performed any adjustment in this study, which makes it difficult to compare results with international data [9]. Another national study was also medical claim-based and reported that the crude incidence of AD increased from 5.63 per 1,000 person-years in 2005 to 8.18 per 1,000 person-years in 2010 among elderly adults aged 50 years and more [14]. However, this study used a closed cohort design merely in 2005 and might only represent the crude incidence of AD from 2005 cohort. In addition, this study reported no age-specific, sex-specific, or age- and sex-specific incidence of AD, which does not allow comparison with estimation from other countries. For prevalence of dementia and AD, although one national survey in Taiwan reported the age-sex-adjusted prevalence of dementia at 8.13 per 10^2^ people (95% CI, 7.61–8.66) among people aged 65 years and older in 2011–2013 [23], there is no national study to investigate the prevalence of AD in Taiwan [24].

Dementia has a complex etiology subject to individual and environmental factors. Older age is the strongest risk factor of dementia [25] and AD [3]. Although the incidence of dementia and AD begins to elevate sharply after 65 years [25–27], a meta-analysis suggested that the incidence of dementia and AD increased slowly or plateaued after age 90 [28]. Two later studies had consistent findings [29, 30]. This means dementia and AD are not natural processes of ageing. In addition, the association of gender with dementia and/or AD risk remains unclear among elderly adults. Previous studies suggested dementia and AD occur more commonly in females, especially in dementia onset at the age of over 85 and AD onset at the age of over 80 [27, 31]. However, a meta-analysis showed no significant differences in the sex-specific incidence and prevalence of dementia [32] and AD [24].

Information on the prevalence and incidence rates of dementia and AD according to demographic factors can help estimate the medical cost and burden of diseases for planning medical services. In Taiwan, the healthy life expectancy is higher than that in the Western countries [33], as well as the potential risk or protective factors for cognition health such as lifestyle patterns and dietary habits are also different from other ethnicities [34]. Thus, we conducted this population-based study using comprehensive claim data from Taiwan's National Health Insurance Research Database to explore whether demographic variations exist in the incidence and prevalence of dementia and AD among people aged 65 years and older.

## 2. Materials and Methods

### 2.1. Data Sources

We obtained data from Taiwan's National Health Insurance Research Database (NHIRD), which is supervised by the National Health Insurance Administration, Ministry of Health and Welfare. A universal National Health Insurance (NHI) program has been implemented in Taiwan since 1995. More than 99% of Taiwan's residents have been enrolled in the NHI program after 1996. The National Health Insurance Administration has contracted with 92.5% of the hospitals and clinics in Taiwan [35] and performs expert reviews quarterly on a random sample in each hospital and clinic to ensure the accuracy of the claims data [35]. Therefore, information obtained from NHIRD is considered to be complete and accurate. We used the NHI medical data of ambulatory care claims, inpatient claims, and and the updated registry for beneficiaries. All datasets were interlinked through each individual's encrypted personal identification number. Access to the NHIRD was approved by the National Health Research Institutes Review Committee on 31 December 2012. When we applied for access to the NHIRD, the most recent data of dementia and AD available were those of 2010; thus, we will report the incidence and prevalence of dementia and AD only in the period between 2004 and 2010. The approved code by the National Health Research Institutes Review Committee is NHIRD-101-565.

### 2.2. Participants

#### 2.2.1. Dementia Study

We identified Taiwanese patients with dementia from the NHIRD, using International Classification of Diseases, Ninth Revision, Clinical Modification (ICD-9-CM) [36], because the ICD-10 was used after 2016. To increase the validity of dementia identification, we included only patients who were diagnosed with dementia with at least three outpatients claim records of dementia-related diagnosis codes (ICD-9-CM codes of 290, 291, 294, 331, and 046.1). This definition was based on the previous studies on dementia using Taiwan's NHI claims [37]. Moreover, to avoid accidental inclusion of miscoded patients, the first and last outpatient visits should be separated by at least 90 days in our study. To include only the first-time diagnosed dementia cases (i.e., incident cases) between 2004 and 2010, we excluded those who obtained a dementia diagnosis before 2004 and those with repeat dementia in the analysis. The date of the first diagnosis of dementia in the period of 2004 to 2010 was set as the index date. Patients who obtained a dementia diagnosis prior to 2004 and those who developed dementia and were alive in the subsequent years after diagnosis of dementia were considered as prevalent cases. With the abovementioned inclusion and exclusion criteria, this study enrolled 181,989 patients with the first-time diagnosed dementia, and 1,196,706 patients with prevalent dementia accumulated from 2004–2010. [Supplementary-material supplementary-material-1] shows the flow chart for the enrollment of participants in the dementia study.

#### 2.2.2. AD Study

We identified patients with AD according to the ICD-9-CM codes of 290.0, 290.1, 290.2, 290.3, or 331.0) [36]. Similar to the method of increasing validity in dementia identification, we included only patients who were regarded as having AD with at least three ambulatory claims with these dementia-related diagnosis codes. The first and last outpatient visits should be separated by at least 90 days to avoid accidental inclusion of miscoded patients. To ensure the validity of AD diagnosis, we excluded those who had 3 or more medical claims (either ambulatory or inpatient care) with diagnostic codes of stroke (ICD-9-CM code: 430-438), drug-induced mental disorders (ICD-9-CM code: 292), alcohol-induced mental disorders (ICD-9-CM code: 291), and Parkinson's disease (ICD-9-CM code: 332) prior to the initial diagnosis of AD. Besides, to include only the first-time diagnosed AD cases (i.e., incident cases) between 2004 and 2010, we excluded those who obtained a dementia-related diagnosis before 2004 and those with repeat dementia in the analysis. The date of the first-time diagnosis of AD in the period of 2004 to 2010 was referred to as the index date. Patients who have developed AD before 2004 and those with AD in the subsequent years after the diagnosis of AD were considered as prevalent cases. With the abovementioned inclusion and exclusion criteria, this study enrolled 83,460 patients with the first-time diagnosed AD, and 560,133 patients with prevalent AD accumulated from 2004–2010. [Supplementary-material supplementary-material-1] shows the flow chart for the enrollment of participants in the AD study.

### 2.3. Demographic Factors

Information on demographic factors, including gender and age, were obtained from the beneficiary records of NHIRD. Gender was divided into male and female. Age at the first-time diagnosis of dementia or AD (for incident case) or at midyear (for prevalent case) was categorized into five age groups: 65–69, 70–74, 75–79, 80–84, and ≥85.

### 2.4. Statistical Methods

We conducted the analyses for the dementia and AD studies separately. The annual crude incidence and prevalence rates were calculated by dividing the number of incident and prevalent cases of dementia or AD, respectively, by the total number of people in the NHI program in each year from 2004 to 2010. We further calculated, using the WHO 2000 standard population, age- and sex-standardized annual incidence and prevalence rates of dementia or AD [38]. Trends of overall standardized incidence/prevalence and age-standardized sex-specific incidence/prevalence were then presented graphically. Additionally, we presented trends of age/sex-specific incidence/prevalence during the study periods. We also calculated the weighted average of the annual incidence/prevalence of age and sex stratifications to present the age- and sex-specific incidence/prevalence rates graphically. Poisson regression was used to test whether there was a linear secular trend in incidence/prevalence of dementia and AD over time. To account for the effects of age, sex, and calendar year on the incidence/prevalence of dementia or AD, we also conducted multivariate Poisson regression analysis. The statistical analysis was performed using SAS version 9.4 (SAS Institute, Cary, NC, USA). A *p* value <0.05 was considered statistically significant.

## 3. Results

**Figure 1:**
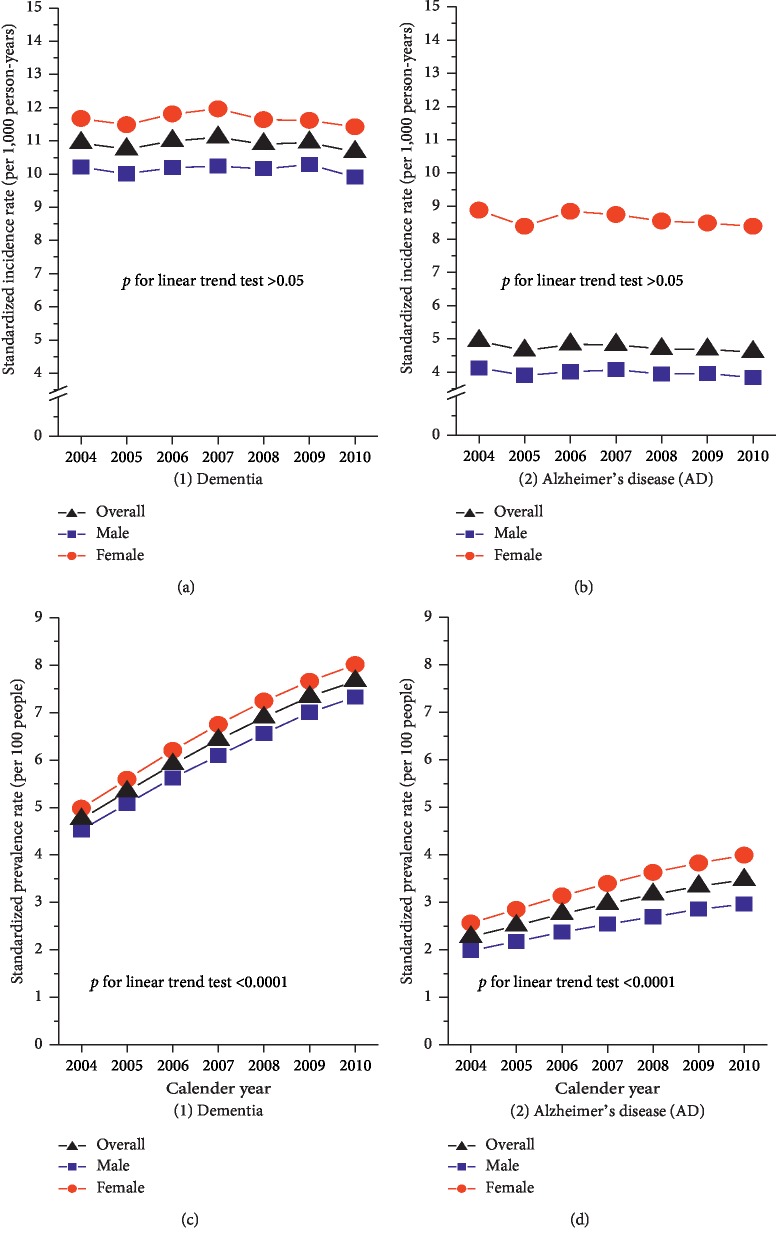
The sex-specific (age-standardized) annual incidence (a, b) and prevalence (c, d) rates of dementia and Alzheimer's disease (AD) in Taiwan, 2004–2010.

The mean age at dementia diagnosis was significantly lower in male than in female (78.4 ± 6.7 vs. 78.7 ± 7.1, *p* < 0.0001), whereas the mean age at AD diagnosis was indifferent between male and female (79.2 ± 6.8 vs. 79.1 ± 7.2, *p*=0.2806).  Table 1 shows that, over the seven years, the age-sex-standardized prevalence of dementia and AD significantly increased from 4.7 to 7.6 per 100 people (*β* = 0.0784, *p* < 0.0001) and from 2.3 to 3.5 per 100 people (*β* = 0.0696, *p* < 0.0001), respectively, representing a 1.63-fold increase for dementia and a 1.54-fold increase for AD. However, during the same period, the incidence of dementia and AD decreased but not significantly from 10.9 to 10.7 per 1,000 person-years (*β* = −0.0012, *p*=0.9437) and from 4.9 to 4.6 per 1,000 person-years (*β* = −0.0069, *p*=0.7480), respectively. Similar secular trends were observed for both male and female. Females consistently had higher incidence and prevalence rates of dementia and AD, with a constant female/male ratio over time (Figure 1).

Tables 2 and 3 illustrate the female/male ratio of prevalence and incidence rates of dementia were 1.1 and 1.1–1.2, and the corresponding figures for AD were 1.3–1.4 and 2.1–2.2, respectively. Over the study period, all increasing trends for prevalence of dementia and AD were statistically significant in any given age group (*p* value for linear trend test <0.0001). For example, regardless of gender, Table 2 indicates that the greatest increase in prevalence of dementia was noted in those aged ≥85 years (male: *β* = 0.1137, *p* < 0.0001; female: *β* = 0.1091, *p* < 0.0001). For both male and female aged ≥85 years, the prevalence of dementia ranged from 13.1 per 100 people in 2004 to 26.6 per 100 people in 2010 and 16.1 per 100 people in 2004 to 31.9 per 100 people in 2010, respectively. As for AD, the highest rise in prevalence of AD for male and female was also observed in those aged ≥85 (male: *β* = 0.0973, *p* < 0.0001; female: *β* = 0.0974, *p* < 0.0001) and ranged from 6.9 per 100 people in 2004 to 12.8 per 100 people in 2010 and 9.4 per 100 people in 2004 to 17.5 per 100 people in 2010, respectively.

Despite this, the secular trend in incidence of dementia and AD varies by age.  Table 3 shows the increased trend in the incidence of dementia after 80 years in male (aged 80–84 years: *β* = 0.0108, *p*=0.0016; aged ≥85 years: *β* = 0.0251, *p* < 0.0001) and 85 years in female (*β* = 0.0116, *p*=0.0008), while the incidence of dementia or AD was stable or declining in other given aged group of both sex. A significant downward trend in incidence of dementia was noted among male aged ≤74 years and among female aged ≤79 years, respectively. The greatest reduction in incidence of dementia was noted in male aged 65–59 years (*β* = −0.0234, *p* < 0.0001) and ranged from 4.3 1,000 person-years in 2004 to 3.6 1,000 person-years in 2010. As for AD, the significant downward trend in incidence of AD was noted among male aged 75–79 years and among female aged ≥75 years, respectively. The greatest decrease in incidence of AD was noted in male aged 75–79 years (*β* = −0.0164, *p*=0.0020) and ranged from 5.4 1,000 person-years in 2004 to 4.6 1,000 person-years in 2010.

**Figure 2:**
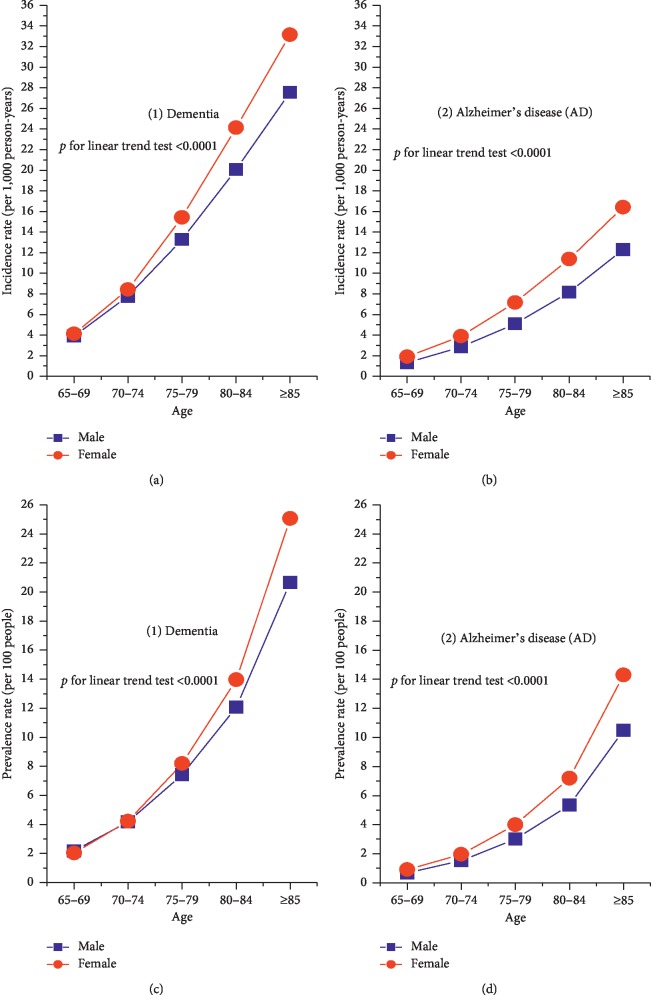
The age- and sex-specific incidence (a, b) and prevalence (c, d) rates of dementia and Alzheimer's disease (AD) in Taiwan, 2004–2010.

Figure 2 also presents that the age-specific incidence was markedly higher in females than in males at the age groups 75–79 through to 85 years and older for dementia and at the age groups 70–74 through to 85 years and older for AD. As to the prevalence rates, the age-specific prevalence started to become markedly higher in females than in males for people with dementia aged 80 years and for people with AD aged 75 years. The gender differences in age-specific incidence and prevalence of dementia and AD was the most evident after age 85 and older. Regardless of gender, the age-specific incidence and prevalence rates of dementia and AD nearly doubled with each 5-year after 65 years. In both gender, there was a positive dose gradient between age and the incidence and prevalence of dementia and AD (*P* for linear trend test <0.0001).

Finally, multivariate Poisson regression models on the incidence and prevalence of dementia and AD are shown in Tables 4 and 5. Compared with that of 2004, the adjusted incidence of dementia revealed a significant decline in 2010 with an adjusted incidence rate ratio (IRR) of 0.98. The adjusted IRR of AD also showed a significant decline in 2005, 2009, and 2010, with corresponding figures ranging from 0.94 to 0.95. However, over the study period, tests for linear trends showed no significant declines in the adjusted IRR of dementia (*β* = −0.0012, *p*=0.9437) and AD (*β* = −0.0069, *p*=0.7480). In contrast, the prevalence of dementia and AD steadily increased in the subsequent years, with adjusted prevalence rate ratio (PRR) of dementia ranging from 1.12 to 1.63 (*p* for overall trend <0.0001) and AD from 1.11 to 1.54 (*p* for overall trend <0.0001).

Age appeared to have more influence on the incidence and prevalence of dementia and AD than gender. Compared with participants aged 65–69 years, very high adjusted IRR (7.58, 95% CI = 7.45–7.71) and adjusted PRR (10.64, 95% CI = 10.56–10.71) of dementia were observed in those aged 85 years and over. The oldest age group (aged ≥85 years) were also associated with significant higher adjusted IRR (8.91, 95% CI = 8.69–9.15) and adjusted PRR (15.04, 95% CI = 14.88–15.20) of AD. There were significant positive trends in both adjusted IRR and PRR across different age groups of dementia and AD (*p* for linear trend test <0.0001). In addition, although there were significant gender variations in the incidence and prevalence of dementia and AD, females had a slightly higher adjusted IRR (dementia: 1.15, 95% CI = 1.14–1.16; AD: 1.38, 95% CI = 1.36–1.40) and adjusted PRR (dementia: 1.11, 95% CI = 1.09–1.12; AD: 1.33, 95% CI = 1.32–1.34) than their male counterparts.

## 4. Discussion

This nationwide study demonstrated significant demographic disparities in the incidence and prevalence of dementia and AD in the Taiwanese population aged 65 years and older. The standardized incidence rate of dementia (10.7–11.1 per 1,000 person-years) noted in this study was lower than the data reported from the previous studies in Europe (17.3 per 1,000 person-years) [2], the United States (17.8 per 1,000 person-years) [2], and Japan (41.6 per 1,000 person-years) [8]. Also, this study showed the standardized prevalence rate of dementia was 7.6 per 10^2^ people in 2010, which is different from the data in Europe (6.8 per 10^2^ people) [2], the United States (8.6 per 10^2^ people) [39], and Japan (11.3 per 10^2^ people) [8]. Similar comparative findings were also observed for the incidence and prevalence rates of AD. Nonetheless, comparisons of the results of our study and with previous ones are difficult, mainly due to the dissimilarities in the source of data (medical claims vs. door-to-door survey), dementia and AD diagnostic criteria, age profile of the study population, and time period of the study.

Our study showed a slight but insignificant decrease in age-sex-standardized incidence of dementia and AD in Taiwan. Some studies from high-income countries such as the United States (1997–2008 [18]) and Netherlands (1992 to 2014) [17] have also reported relatively stable trends in the incidence of dementia [17] or AD [18]. Other studies from the Germany (2006-2007 to 2009–2010) [20] and Canada (2005-2006 to 2012-2013) [6] have even reported decreasing trends in the incidence of dementia, while other studies from Japan (1988 and 2012) [8] and Taiwan (1996–2003) [9] demonstrated an increasing trend.

As for AD, a recent Taiwanese study has showed an increasing trend in AD incidence among people aged 50 years and older from 2005 to 2010 [14], which is inconsistent with our findings. Inconsistency in the secular trends in the incidence of dementia and AD between ours and prior studies could be due to differences in the age profile of study participants, the time period of the study, the criteria of dementia, the diagnostic technique for dementia, or the protective/risk factors for dementia [6, 8, 9, 12–14, 16–20]. In addition, a study in Beijing, China, indicated a slightly increasing trend in the incidence of dementia might be due to the change in diagnostic criteria and the improvement in diagnosis of dementia [12]. As to the study years, unlike our study that included only people aged 65 years and older between 2004 and 2010, previous Taiwanese studies mentioned above included people aged 65 years and older from 1996 to 2003 [9] or people aged 50 years and older between 2005 and 2010 [14].

Regarding factors associated with dementia, the previous studies suggested that ageing, saturated fats, smoking, physical inactivity, and preexisting medical diseases such as cardiovascular disease could increase the risk of developing dementia and AD [3]. A stable trend in the age-sex-standardized incidence of dementia and AD noted in our study could be partly due to reduced smoking [40] and improvements in vascular health [41] over time. National Health Interview Surveys in Taiwan showed the prevalence of smoking significantly declined in males from 44.4% in 2001 to 34.2% in 2013, while the corresponding figures being stable in females during the same period [40]. Another study by Yang et al. reported the hospitalization rate of acute myocardial infarction in Taiwan significantly decreased from 0.35‰ to 0.06‰ between 1999 and 2008, which may partly reflect a successful improvement in vascular health [41]. Thus, we also suspected that an increased trend in the incidence of dementia after 80 years in male and 85 years in female, which may result from more and more people live longer and are healthier in their life. These conditions compress their onset of dementia to the older age.

We found a gradual rise in the prevalence of dementia, which is in accordance with findings from other studies in France (1998–2008) [5], Canada (2005–2013) [6], Japan (1985–2005 [7] and 1985–2012 [8]), mainland China, Hong Kong, and Taiwan (pre-1990 and post-2010) [11]. According to the WHO report, with continuing declines in death rates among older people, we speculate that the prevalence of dementia rises as the life expectancy increasing among elderly population [42].

Inconsistent findings have been disclosed concerning the gender differences in the incidence of dementia and AD [24, 27, 31, 32, 43, 44]. Many studies noted females had higher incidence rates of dementia and AD than males, especially in the oldest age groups [27, 31], while other studies demonstrated no significant gender differences [24, 32]. Our study showed females were more likely than males to have a diagnosis of dementia (AIRR = 1.15, 95% CI = 1.14–1.16) or AD (AIRR = 1.38, 95% CI = 1.36–1.40), which was close to figures (2 fold) noted in some previous studies [43, 44].

Additionally, our study showed gender differences increased with age and began after 75 years for people suffering from dementia or after 70 years for people suffering from AD. Some previous studies have also found a higher incidence of dementia and AD in females, especially in those suffering from dementia at age 85 or those suffering from AD at age 80 [27, 31], which may be partially explained by the neuroprotection effects of estrogen among females until menopause [45]. Other explanations for gender disparity in the risk of dementia and AD are mainly due to lower educational attainment [46], a stronger Apolipoprotein E genotype effect [46], smaller head size [47], or slower cerebral brain volume [47] in females. It is also hypothesized that the longer female life expectancy may be another possible interpretation with respect to the higher incidence of dementia and AD among females [48]. Moreover, our study findings were in accordance with previous study results suggesting the higher prevalence of dementia and AD in females. This may be due to the higher incidence of dementia and AD in females or the shorter male survival time after diagnosis of dementia and AD.

### 4.1. Contributions

To the best of our knowledge, this is the first nationwide population-based study in Taiwan covering people aged 65 years and older, exploring the secular trends in incidence and prevalence of dementia and AD. We obtained a large number of participants by using NHI claims data, which made it possible to conduct highly representative age and sex analyses.

### 4.2. Limitations

This study has the following limitations. First, although we selected dementia and AD patients according to physician-recorded diagnosis, it might still lead to disease misclassification of dementia and AD. To avoid accidental inclusion of miscoded patients, we included solely dementia and AD cases that had at least 3 outpatient visits with dementia-related diagnosis and the first and last visits more than 90 days apart during the study period. Second, by using NHI claim data, we included only the patients diagnosed with dementia in hospitals or clinics. This may underestimate the incidence and prevalence of dementia with the possibility that elderly subjects may have been diagnosed and treated with other comorbid diseases instead of being diagnosed with dementia. Third, due to the limited information available from the claims data, several potential confounders that may influence the incidence and prevalence of dementia and AD were not included in the analyses. These potential confounders include smoking, educational level, occupation, physical function, and specific environmental features.

## 5. Conclusions

In conclusion, despite a stable trend in the incidence of dementia and AD from 2004 to 2010 in Taiwan, the age-sex- adjusted prevalence of dementia and AD substantially increased over the study period. Further studies are warranted to explore factors associated with the incidence and prevalence of dementia and AD by different gender and age groups. Also, an intervention program for managing dementia and AD considering demography disparity is recommended.

## Figures and Tables

**Table 1:** Secular trends of overall incidence and prevalence rates of dementia and Alzheimer's disease (AD) in Taiwan, 2004–2010.

Variable	Incidence (per 1,000 person-years)^‡^							Change (%)^†^	Trend test *β*	*p*	Prevalence rate (per 100 people)^‡^							Change (%)^†^	Trend test *β*	*p*
	Calendar year										Calendar year									
	2004	2005	2006	2007	2008	2009	2010				2004	2005	2006	2007	2008	2009	2010			
*Dementia*																				
Crude	**11.2**	**11.2**	**11.6**	**11.8**	**11.7**	**11.9**	**11.8**	6.2	0.0105^*∗*^	<0.0001	5.0	**5.7**	**6.4**	**7.1**	**7.8**	**8.4**	**9.1**	82.0	0.0972^*∗*^	<0.0001
Adjusted^‡^	**10.9**	**10.7**	**11.0**	**11.1**	**10.9**	**10.9**	**10.7**	−1.8	−0.0012	0.9437	4.7	**5.3**	**5.9**	**6.4**	**6.9**	**7.3**	**7.6**	61.7	0.0784^*∗*^	<0.0001

*AD*																				
Crude	**5.1**	**4.9**	**5.1**	**5.2**	**5.2**	**5.2**	**5.3**	3.9	0.0076^*∗*^	<0.0001	**2.4**	**2.7**	**3.0**	**3.3**	**3.6**	**3.9**	**4.2**	75.0	0.0920^*∗*^	<0.0001
Adjusted^‡^	**4.9**	**4.6**	**4.8**	**4.8**	**4.7**	**4.7**	**4.6**	−6.1	−0.0069	0.7480	**2.3**	**2.5**	**2.8**	**3.0**	**3.2**	**3.3**	**3.5**	52.1	0.0696^*∗*^	<0.0001

^‡^Adjusted for age and sex. ^†^Change (%): percentage of changes in the incidence and prevalence rates of dementia or Alzheimer's disease AD between 2004 and 2010. ^*∗*^*p* for linear trend test <0.05.

**Table 2:** Age- and sex-specific prevalence rate^‡^ of dementia and Alzheimer's disease (AD) and secular trend in Taiwan, 2004–2010.

Sex and age	Calendar year							Change^†^ (%)	Trend test *β*	*p*
	2004	2005	2006	2007	2008	2009	2010			
*Dementia*										
Male										
65–69	1.8	1.9	2.1	2.2	2.3	2.4	2.6	44.4	0.0563^*∗*^	<0.0001
70–74	3.4	3.7	4.1	4.3	4.5	4.6	4.6	35.3	0.0498^*∗*^	<0.0001
75–79	5.7	6.4	7.0	7.6	8.1	8.6	8.8	54.4	0.0711^*∗*^	<0.0001
80–84	8.7	9.9	10.9	11.9	12.9	13.9	14.6	67.8	0.0829^*∗*^	<0.0001
≥85	13.1	15.3	17.6	19.7	21.9	24.6	26.6	103.1	0.1137^*∗*^	<0.0001
Total^§^	4.5	5.1	5.6	6.1	6.6	7.0	7.3	62.2	0.0785^*∗*^	<0.0001
Female										
65–69	1.6	1.8	2.0	2.1	2.2	2.3	2.4	50.0	0.0584^*∗*^	<0.0001
70–74	3.4	3.7	4.0	4.3	4.5	4.6	4.8	41.2	0.0563^*∗*^	<0.0001
75–79	6.3	7.1	7.7	8.3	8.8	9.2	9.3	47.6	0.0619^*∗*^	<0.0001
80–84	10.4	11.6	12.8	13.9	15.0	15.8	16.5	58.7	0.0748^*∗*^	<0.0001
≥85	16.1	18.9	21.6	24.3	27.0	29.7	31.9	98.1	0.1091^*∗*^	<0.0001
Total^§^	5.0	5.6	6.2	6.8	7.2	7.7	8.0	60.0	0.0772^*∗*^	<0.0001
Female/male ratio	1.1	1.1	1.1	1.1	1.1	1.1	1.1			

*AD*										
Male										
65–69	0.6	0.6	0.7	0.7	0.7	0.8	0.8	33.3	0.0465^*∗*^	<0.0001
70–74	1.3	1.4	1.5	1.6	1.6	1.6	1.6	23.1	0.0349^*∗*^	<0.0001
75–79	2.4	2.7	2.9	3.1	3.2	3.4	3.4	41.7	0.0546^*∗*^	<0.0001
80–84	4.2	4.6	4.9	5.3	5.6	6.0	6.2	47.6	0.0646^*∗*^	<0.0001
≥85	6.9	8.0	9.1	9.9	10.9	12.0	12.8	85.5	0.0973^*∗*^	<0.0001
Total^§^	2.0	2.2	2.4	2.5	2.7	2.9	3.0	50.0	0.0658^*∗*^	<0.0001
Female										
65–69	0.7	0.8	0.9	0.9	1.0	1.0	1.1	57.1	0.0639^*∗*^	<0.0001
70–74	1.6	1.7	1.9	2.0	2.1	2.1	2.2	37.5	0.0561^*∗*^	<0.0001
75–79	3.2	3.5	3.8	4.1	4.3	4.4	4.5	40.6	0.0575^*∗*^	<0.0001
80–84	5.6	6.1	6.7	7.1	7.6	7.9	8.2	46.4	0.0630^*∗*^	<0.0001
≥85	9.4	11.0	12.4	13.8	15.1	16.4	17.5	86.2	0.0974^*∗*^	<0.0001
Total^§^	2.6	2.8	3.1	3.4	3.6	3.8	4.0	53.8	0.0723^*∗*^	<0.0001
Female/male ratio	1.3	1.3	1.3	1.3	1.4	1.3	1.4			

^‡^Per 100 people. ^§^Adjusted for age. ^†^Change (%): percentage of changes in the prevalence rates of dementia or AD between 2004 and 2010. ^*∗*^*p* for linear trend test <0.05.

**Table 3:** Age- and sex-specific incidence density‡ of dementia and Alzheimer's disease (AD) and secular trend in Taiwan, 2004–2010.

Sex and age	Calendar year							Change^†^ (%)	Trend test *β*	*p*
	2004	2005	2006	2007	2008	2009	2010			
*Dementia*										
Male										
65–69	4.3	4.1	4.0	3.8	3.9	3.8	3.6	−16.2	−0.0234^*∗*^	<0.0001
70–74	8.2	8.0	7.9	8.0	7.7	7.4	7.2	−12.2	−0.0195^*∗*^	<0.0001
75–79	13.1	13.3	13.4	13.7	13.5	13.3	12.5	−4.6	−0.0047	0.1622
80–84	18.9	19.6	20.1	20.4	20.2	21.1	20.0	5.8	0.0108^*∗*^	0.0016
≥85	26.7	24.7	27.0	27.0	26.9	29.6	29.6	10.9	0.0251^*∗*^	<0.0001
Total^§^	10.2	10.0	10.2	10.2	10.2	10.3	9.9	−2.9	−0.0013	0.9497
Female										
65–69	4.2	4.1	4.3	4.2	4.2	4.0	3.9	−7.1	−0.0129^*∗*^	0.0077
70–74	8.8	8.5	8.5	8.7	8.2	8.0	8.3	−5.7	−0.0111^*∗*^	0.0029
75–79	15.4	15.5	15.7	15.9	15.4	15.4	14.7	−4.5	−0.0069^*∗*^	0.0325
80–84	23.8	23.4	24.0	25.0	24.5	24.7	23.6	−0.8	0.0034	0.2975
≥85	32.1	31.3	33.5	33.3	33.1	33.8	34.2	6.5	0.0116^*∗*^	0.0008
Total^§^	11.7	11.5	11.8	12.0	11.6	11.6	11.4	−2.6	−0.0020	0.9167
Female/male ratio	1.1	1.2	1.2	1.2	1.2	1.1	1.2			

*AD*										
Male										
65–69	1.4	1.3	1.3	1.3	1.3	1.3	1.3	−7.1	−0.0064	0.4724
70–74	3.0	2.7	2.9	3.0	2.8	2.8	2.7	−10.0	−0.0111	0.0877
75–79	5.4	5.1	5.1	5.3	5.2	5.0	4.6	−14.8	−0.0164^*∗*^	0.0020
80–84	8.0	8.1	8.1	8.5	8.3	8.3	7.9	−1.3	0.0006	0.9142
≥85	12.9	11.8	13.0	12.0	11.6	12.4	12.6	−2.3	−0.0021	0.7168
Total^§^	4.1	3.9	4.0	4.1	3.9	4.0	3.8	−7.3	−0.0074	0.8230
Female										
65–69	2.0	1.9	1.9	1.9	1.8	1.9	1.9	−5.0	−0.0053	0.4546
70–74	3.9	3.7	4.0	3.9	3.9	3.8	4.0	2.6	0.0026	0.6254
75–79	7.3	7.1	7.4	7.4	7.2	7.1	6.7	−8.2	−0.0112^*∗*^	0.0162
80–84	11.9	11.1	11.6	11.7	11.4	11.1	10.9	−8.4	−0.0101^*∗*^	0.0300
≥85	17.9	16.4	16.8	16.5	15.9	15.9	16.1	−10.1	−0.0155^*∗*^	0.0010
Total^§^	8.9	8.4	8.8	8.7	8.6	8.5	8.4	−5.6	−0.0064	0.7767
Female/male ratio	2.2	2.2	2.2	2.2	2.2	2.1	2.2			

^‡^Per 1,000 person-years.^§^Adjusted for age. ^†^Change (%): percentage of changes in the incidence rates of dementia or AD between 2004 and 2010. ^*∗*^*p* for linear trend test <0.05.

**Table 4:** Multivariate Poisson regression model on the incidence and prevalence rates of dementia in Taiwan from 2004–2010.

Variables	Incidence rates				Prevalence rates			
	Crude IRR^†^	95% CI^†^	Adjusted IRR^†^	95% CI^†^	Crude PRR^†^	95% CI^†^	Adjusted PRR^†^	95% CI^†^
*Year (ref.* *=* *2004)*^†^								
2005	0.99	0.98–1.01	0.98	0.96–1.00	1.14^*∗*^	1.13–1.15	1.12^*∗*^	1.11–1.13
2006	1.03^*∗*^	1.01–1.05	1.01	0.99–1.02	1.29^*∗*^	1.28–1.30	1.24^*∗*^	1.23–1.25
2007	1.05^*∗*^	1.03–1.07	1.02	0.98–1.02	1.43^*∗*^	1.42–1.43	1.35^*∗*^	1.34–1.36
2008	1.04^*∗*^	1.02–1.06	1.00	1.00–1.03	1.56^*∗*^	1.55–1.57	1.45^*∗*^	1.44–1.46
2009	1.06^*∗*^	1.04–1.08	1.01	0.99–1.02	1.69^*∗*^	1.68–1.70	1.55^*∗*^	1.54–1.56
2010	1.05^*∗*^	1.04–1.07	0.98^*∗*^	0.97–1.00	1.82^*∗*^	1.81–1.84	1.63^*∗*^	1.61–1.64
Trend test	*β* = 0.0105, *p* < 0.0001		*β* = −0.0012, *p*=0.9437		*β* = 0.0972, *p* < 0.0001		*β* = 0.0784, *p* < 0.0001	

*Age (ref.* *=* *65–69)*^†^								
70–74	2.01^*∗*^	1.98–2.05	2.01^*∗*^	1.98–2.05	1.99^*∗*^	1.97–2.20	1.98^*∗*^	1.97–2.00
75–79	3.55^*∗*^	3.50–3.61	3.58^*∗*^	3.52–3.64	3.69^*∗*^	3.66–3.71	3.71^*∗*^	3.68–3.73
80–84	5.46^*∗*^	5.37–5.55	5.51^*∗*^	5.42–5.60	6.14^*∗*^	6.09–6.18	6.08^*∗*^	6.04–6.12
≥85	7.58^*∗*^	7.45–7.71	7.58^*∗*^	7.45–7.71	10.88^*∗*^	10.81–10.96	10.64^*∗*^	10.56–10.71
Trend test	*β* = 0.4907, *p* < 0.0001		*β* = 0.4913, *p* < 0.0001		*β* = 0.5799, *p* < 0.0001		*β* = 0.5732, *p* < 0.0001	

*Sex (ref.* *=* *male)*^†^								
Female	1.11^*∗*^	1.10–1.12	1.15^*∗*^	1.14–1.16	1.10^*∗*^	1.09–1.11	1.11^*∗*^	1.09–1.12

^†^IRR, incidence rate ratio; PRR, prevalence rate ratio; CI, confidence interval; Ref., reference. ^*∗*^*p* < 0.05.

**Table 5:** Multivariate Poisson regression model on incidence and prevalence rates of Alzheimer's disease (AD) in Taiwan from 2004–2010.

Variables	Incidence rates				Prevalence rates			
	Crude IRR^†^	95% CI^†^	Adjusted IRR^†^	95% CI^†^	Crude PRR^†^	95% CI^†^	Adjusted PRR^†^	95% CI^†^
*Year (ref.* *=* *2004)*^†^								
2005	0.96^*∗*^	0.93–0.98	0.94^*∗*^	0.92–0.97	1.13^*∗*^	1.11–1.14	1.11^*∗*^	1.09–1.12
2006	1.01	0.98–1.03	0.98	0.95–1.00	1.26^*∗*^	1.25–1.28	1.21^*∗*^	1.19–1.22
2007	1.02	1.00–1.05	0.98	0.95–1.00	1.39^*∗*^	1.38–1.41	1.30^*∗*^	1.29–1.32
2008	1.01	0.98–1.03	0.95	0.93–1.00	1.52^*∗*^	1.50–1.53	1.39^*∗*^	1.38–1.41
2009	1.02	0.99–1.05	0.95^*∗*^	0.92–0.97	1.63^*∗*^	1.62–1.65	1.47^*∗*^	1.46–1.49
2010	1.03^*∗*^	1.01–1.05	0.94^*∗*^	0.91–0.96	1.76^*∗*^	1.74–1.78	1.54^*∗*^	1.52–1.55
Trend test	*β* = 0.0076, *p* < 0.0001		*β* = −0.0069, *p*=0.7480		*β* = 0.0920, *p* < 0.0001		*β* = 0.0696, *p* < 0.0001	

*Age (ref.* *=* *65–69)*^†^								
70–74	2.09^*∗*^	2.03–2.15	2.09^*∗*^	2.04–2.15	2.18^*∗*^	2.15–2.20	2.17^*∗*^	2.15–2.20
75–79	3.74^*∗*^	3.65–3.84	3.80^*∗*^	3.70–3.90	4.34^*∗*^	4.29–4.39	4.39^*∗*^	4.34–4.44
80–84	5.96^*∗*^	5.81–6.11	6.07^*∗*^	5.92–6.23	7.72^*∗*^	7.63–7.80	7.73^*∗*^	7.65–7.82
≥85	8.90^*∗*^	8.68–9.14	8.91^*∗*^	8.69–9.15	15.38^*∗*^	15.21–15.54	15.04^*∗*^	14.88–15.20
Trend test	*β* = 0.5242, *p* < 0.0001		*β* = 0.5255, *p* < 0.0001		*β* = 0.6621, *p* < 0.0001		*β* = 0.6553, *p* < 0.0001	

*Sex (ref.* *=* *male)*^†^								
Female	1.34^*∗*^	1.32–1.36	1.38^*∗*^	1.36–1.40	1.32^*∗*^	1.31–1.33	1.33^*∗*^	1.32–1.34

^†^IRR, incidence rate ratio; PRR, prevalence rate ratio; CI, confidence interval; Ref., reference. ^*∗*^*p* < 0.05.

## Data Availability

Raw data sharing from National Health Insurance Research Database is prohibited according to the National Health Research Institutes policies in Taiwan.

## Supplementary Materials

Supplementary Materials Figure S1: flow chart for participant enrollment of the dementia study; Figure S2: flow chart for participant enrollment of Alzheimer's disease (AD) study.
