# Supplementary material for: Gender and Age Differences and the Trend in the Incidence and Prevalence of Dementia and Alzheimer's Disease in Taiwan: A 7-Year National Population-Based Study
Source: Biomed Res Int. 2019 Nov 11;2019:5378540. doi: 10.1155/2019/5378540 (PMC6878786; doi:10.1155/2019/5378540)
Supplement: Supplementary Materials — Figure S1: flow chart for participant enrollment of the dementia study; Figure S2: flow chart for participant enrollment of Alzheimer's disease (AD) study. [file 5378540.f1.pdf]

Supplementary Materials:

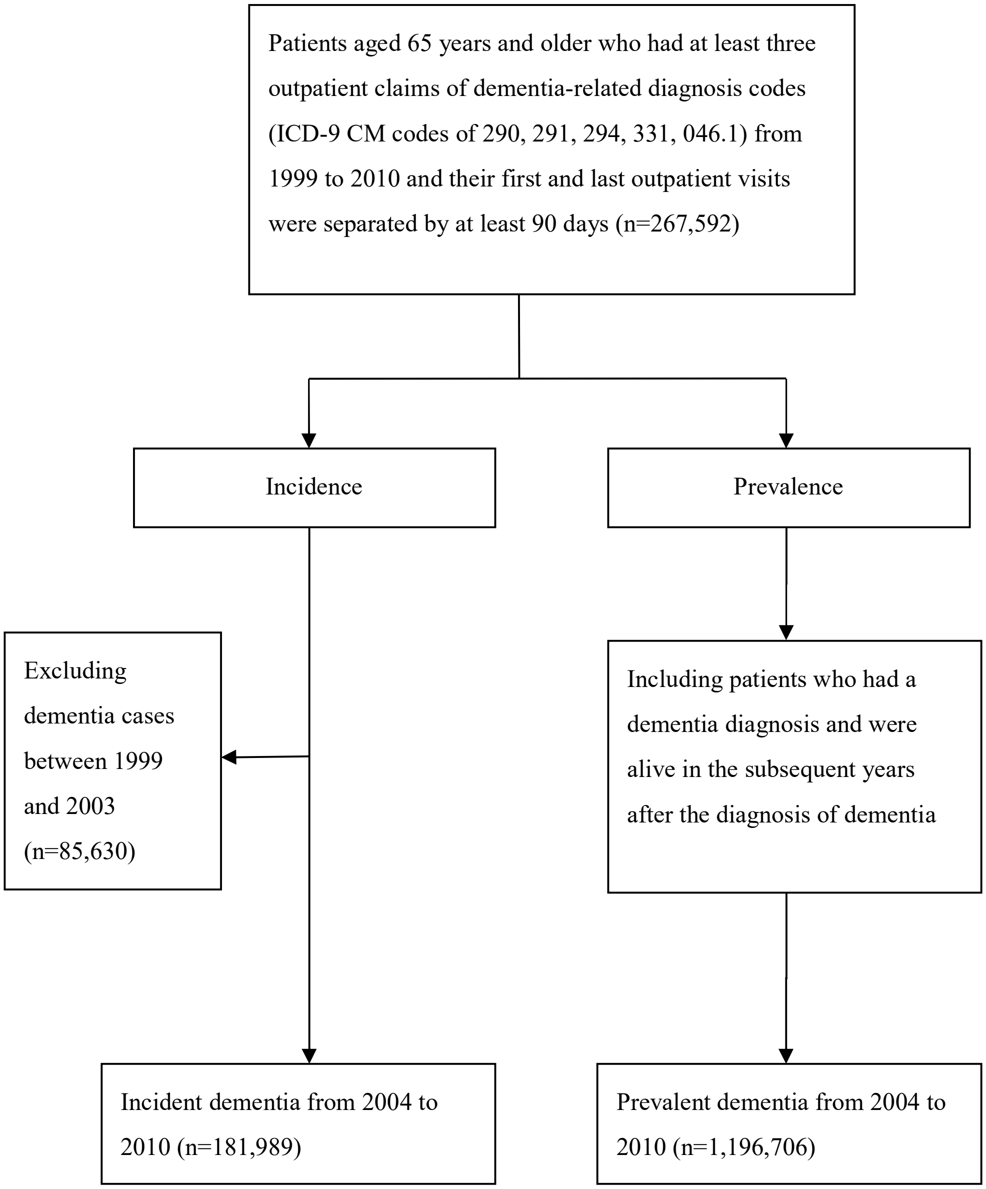

Figure S1: Flow chart for participant enrollment of the dementia study.

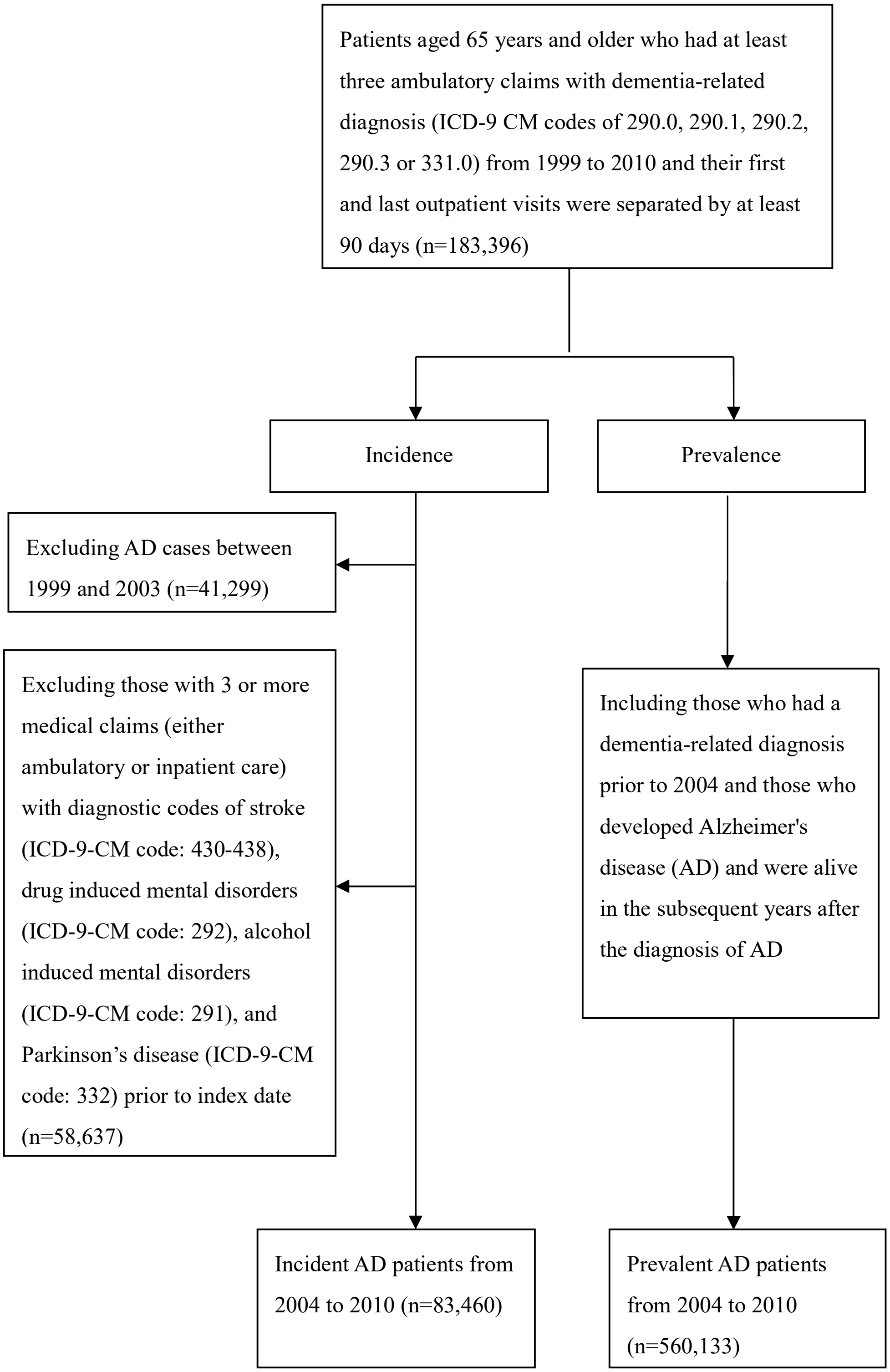

Figure S2: Flow chart for participant enrollment of the Alzheimer's disease (AD) study.
